# *Spatholobus suberectus* inhibits lipogenesis and tumorigenesis in triple-negative breast cancer via activation of AMPK-ACC and K-Ras-ERK signaling pathway

**DOI:** 10.1016/j.jtcme.2023.09.002

**Published:** 2023-09-13

**Authors:** Xiaohui Zeng, Guowei Gong, Kumar Ganesan, Yi Wen, Qingqing Liu, Juncheng Zhuo, Jianming Wu, Jianping Chen

**Affiliations:** a School of Chinese Medicine, Li Ka Shing Faculty of Medicine, The University of Hong Kong, Sassoon Road, Pokfulam, Hong Kong, China; b Guangdong Second Traditional Chinese Medicine Hospital, Guangdong Province Engineering Technology Research Institute of Traditional Chinese Medicine, Guangzhou, Guangdong, 510095, China; c Zhongshan People's Hospital, 106, Zhongshan 2nd Road, Guangdong Province, 510080, China; d School of Pharmacy, Southwest Medical University, Luzhou, 646000, China; e Shenzhen Institute of Research and Innovation, The University of Hong Kong, Shenzhen, 518000, China

**Keywords:** *Spatholobi suberectus*, Lipogenesis, TNBC, Therapy, Natural product, Proteomic and metabolomic analysis

## Abstract

**Background and aim:**

Triple-negative breast cancer (TNBC) is a highly invasive type of breast cancer with a poor prognosis. Currently, there are no effective management strategies for TNBC. Earlier, our lab reported the percolation of *Spatholobus suberectus* for the treatment of breast cancer. Lipid metabolic reprogramming is a hallmark of cancer. However, the anti-TNBC efficiency of *S. suberectus* extract and its causal mechanism for preventing lipogenesis have not been fully recognized. Hence, the present study aimed to investigate the inhibitory role of *S. suberectus* extract on lipogenesis and tumorigenesis in TNBC *in vitro* and *in vivo* by activating AMPK-ACC and K-Ras-ERK signaling pathways using lipidomic and metabolomic techniques.

**Experimental procedure:**

Dried stems of *S. suberectus* extract inhibited lipogenesis and tumorigenesis and promoted fatty acid oxidation as demonstrated by the identification of the metabolites and fatty acid markers using proteomic and metabolomic analysis, qPCR, and Western blot.

**Results and conclusion:**

The results indicated that *S. suberectus* extract promotes fatty acid oxidation and suppresses lipogenic metabolites and biomarkers, thereby preventing tumorigenesis via the AMPK-ACC and K-Ras-ERK signaling pathways. On the basis of this preclinical evidence, we suggest that this study represents a milestone and complements Chinese medicine. Further studies remain underway in our laboratory to elucidate the active principles of *S. suberectus* extract. This study suggests that *S. suberectus* extract could be a promising therapy for TNBC.

## Highlights

• SSP inhibits lipogenesis and tumorigenesis and promotes fatty acid oxidation.
• It is determined by proteomic and metabolomic analysis.
• SSP inhibits tumorigenesis via the AMPK-ACC and K-Ras-ERK signaling pathways.

## Graphical abstract

**Figure:**
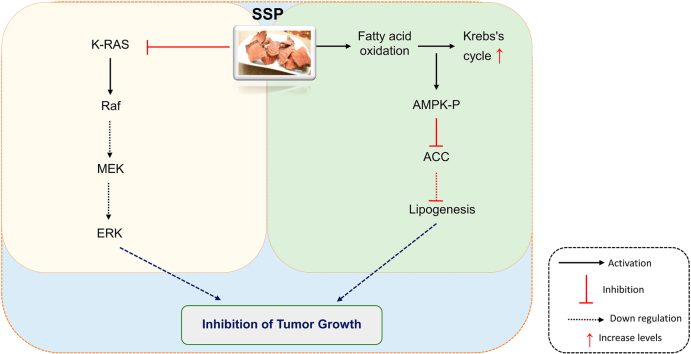

## Abbreviations

5-HT: 5-hydroxytryptamine
5-HTP: 5-hydroxytryptophan
AA: acotinic acid
ACC: acetyl-CoA carboxylase
Akt: protein kinase B
ALT: alanine transaminase
AMPK: 5′ AMP-activated protein kinase
AST: aspartate transaminase
BUN: blood urea nitrogen
CA: citric acid
cDNA: complementary deoxyribonucleic acid
CPT1: carnitine palmitoyltransferase
CREA: creatinine
CS: citrate synthase
CV: Coefficient of variation
CYP7A1: cholesterol 7 alpha-hydroxylase
DGAT: diacylglycerol acyltransferase
DMEM: Dulbecco's Modified Eagle Medium
EGFR: epidermal growth factor receptor
ERK: extracellular signal-related kinase
FA: fatty acid
FAS: fatty acid synthase
FBS: fetal bovine serum
FMA: fumaric acid
G6Pase: glucose 6- phosphatase
GAPDH: glyceraldehyde-3-phosphate dehydrogenase
GCDA: glycochenodeoxycholate
HK: hexokinase
HMGCR: 3-Hydroxy-3-Methylglutaryl-CoA Reductase
HSL: hormone sensitive lipase
IDHα: isocitrate dehydrogenase
iTRAQ: isobaric tags for relative and absolute quantitation
KEGG: Kyoto encyclopedia of genes and genomes
Kyn: l-Kynurenine
LC-MS/MS: Liquid Chromatography Tandem Mass Spectrometry
LDHα: lactate dehydrogenease
LPL: lipoprotein lipase
MAPK: mitogen-activated protein kinase
MDH: malate dehydrogenase
MeOH: methanol
MTS: 3-(4,5-dimethylthiazol-2-yl)-5-(3-carboxymethoxyphenyl)-2-(4-sulfophenyl)-2H-tetrazolium)
NEM: ethylmaleimide
PBS: phosphate buffer saline
PDHC: pyruvate dehydrogenase complex
PFA: paraformaldehyde
Phe-d5: l-phenylalanine-d5
PI3k: phosphatidylinositol 3-kinase
PLS-DA: partial least square discriminant analysis
qPCR: quantitative polymerase chain reaction
RAS: rat sarcoma gene
ROS: reactive oxygen species
RPMI: Roswell Park Memorial Institute
SD: standard deviation
SMPDB: Small molecule pathway database
SREBP1: sterol regulatory element-binding protein 1
SSD: Spatholobus suberectus Dunn
SSP: percolation of Spatholobus suberectus
TCM: traditional Chinese medicine
TNBC: triple-negative breast cancer
Try: L-tryptophan

## 1 Introduction

According to the most recent WHO data, the incidence of breast cancer (BC) has surpassed that of lung cancer and has become the most common cancer in the world.^1^ Western medicine offers standard treatment for BC; however, it has certain limitations. Relapse after surgery,^2^^,^^3^ side effects of radiotherapy and chemotherapy,^4^ drug resistance to chemotherapy,^5^^,^^6^ and lack of effective strategies for treating triple-negative breast cancer (TNBC)^7^^,^^8^ are significant problems associated with TNBC. TNBC is associated with high histological grade, an aggressive phenotype, and poor prognosis. They account for approximately 15% of all invasive BC cases, and have the greatest rate of metastatic incidence and the poorest overall survival of all BC subtypes. Despite the advantage of standard chemotherapy for patients with TNBC, they still have high recurrence rates and are likely to develop resistance to chemotherapy agents.^9^ Furthermore, recent studies have report that deregulation of fatty acid (FA) metabolism is also highly associated with the development of TNBC.^10^^,^^11^

Lipogenesis and its elevation have been associated with augmented cancer risk and poor prognosis. Cancer cells exhibit a high need for glucose, glutamine, and lipids, which are essential for their unrestrained proliferation.^12^ Changes in lipid metabolism, such as elevated lipogenesis and elevated FA uptake, play key roles in BC development, as these BC cells are frequently encircled by large adipocytes that dynamically initiate the triglyceride cycle and maintain an FA-rich milieu.^13^ Through β-oxidation, FAs generate energy and provide construction for phospholipids, which play an extrinsic role in tumor formation.^14^ Recent studies have confirmed that increased lipogenesis is crucial for self-renewal and BC stem cell development, which is recognized as a hallmark of malignancy.^15^ Thus, there is a serious requirement to explore the susceptibility of TNBC and develop novel beneficial agents to improve clinical results for patients with TNBC. Considering the extensive clinical experience in treating tumors, traditional Chinese medicine (TCM) is a valuable opportunity to find solutions to the these problems.

*Spatholobi caulis*, the vine stem of *Spatholobus suberectus* Dunn (SSD, Family: Leguminosae) is a characteristic of TCM used to treat rheumatism, irregular menstruation, dysmenorrhea, amenorrhea, rheumatic arthralgia, numbness, paralysis, anemia, menoxenia, revitalizing blood circulation and eradicating stasis. *S. suberectus* has numerous pharmacological properties, including antibacterial, anti-inflammatory, antimutagenic, antioxidant, antiplatelet, antiviral, stimulating blood circulation, and neuroprotection.^16^, ^17^, ^18^, ^19^, ^20^, ^21^ Previously, *S. suberectus* was found to be anti-TNBC both *in vitro* and *in vivo*, after measuring cell viability, cell cycle, morphological changes, LDH release, ROS generation, DNA fragmentation assay, mitochondrial membrane potential assay, and glutathione aborted the pyroptotic non-inflammasome signaling pathway.^9^^,^^22^^,^^23^ In addition, several experimental studies have reported that crude extracts and active compounds obtained from *S. suberectus* may also be effective in BC management based on possible molecular mechanisms.^24^, ^25^, ^26^, ^27^, ^28^, ^29^ *S. suberectus* encompasses many active compounds in which flavonoids are principal compounds, including procyanidin B2, epicatechin, genistein, formononetin, 3′,4′,7-trihydroxyflavone, 3′-hydroxy-8-methoxyvestitol, butin, calycosin, dihydrokaempferol, dihydroquercetin, eriodictyol, liquiritigenin, prunetin and plathymenin, and most of them exert anticancer properties.^9^^,^^20^^,^^26^^,^^30^ Epigallocatechin gallate is the major component of *S. suberectus* that inhibits BC cell proliferation by inhibiting LDH-A through mediating dissociating HIF-1α from Hsp90 ^23^. TCM practitioners have also successfully used *S. suberectus* to treat BC patients in the clinic.^28^

Proteomics provides disease-specific molecular targets for treatment and prognosis through bioinformatics analysis of large datasets.^31^ Owing to its high quantitative precision, proteomics has been extensively employed in the study of drug discovery studies.^32^ It can simultaneously measure up to eight samples using isobaric tags for relative and absolute quantitation (iTRAQ). Thus, it has been widely used to explore drug mechanisms.^33^ Metabolomics can provide the metabolic profile of mice, which may aid in determining the disease status in the body.^34^^,^^35^

Based on the results of our earlier studies, *S. suberectus* exhibits potent anti-TNBC effects on BC with the potential to cause apoptosis and inhibit cell cycle, LDH formation, and BC cell invasion through the PI3K/AKT/MAPK pathway.^22^^,^^27^ Nevertheless, the anti-TNBC effects of *S. suberectus* and its causal mechanism through inhibition of lipogenesis have not been fully explored. Hence, the present study aimed to explore the preventive role of *S. suberectus* on lipogenesis and tumorigenesis in TNBC via the activation of the AMPK-ACC and K-Ras-ERK signaling pathways.

## 2 Materials and methods

### 2.1 Chemicals and apparatus

l-Kynurenine (257478, Kyn), l-tryptophan (Try), 5-hydroxytryptamine (M110, 5-HT), and 5-hydroxytryptophan (252999, 5-HTP), penicillin and streptomycin (Sigma-Aldrich, St. Louis, USA); l-phenylalanine-d5 (56253-90-8, Phe-d5, Santa Cruz Biotechnology, Inc. Acetonitrile (HPLC grade, Merck, USA); Roswell Park Memorial Institute (RPMI) 1640, fetal bovine serum (FBS) and Dulbecco's Modified Eagle Medium (DMEM) (Gibco, Grand Island, NY, USA); Accu-Chek Active test strips (Roche Diabetes Care GmbH, Co. Australia); TRIzol® reagent (Invitrogen Life Technologies, Carlsbad, CA, USA); Millipore Express PES Membrane (CHVE01TS3, Merck Millipore Ltd., Germany); formic acid (HPLC grade, Acros USA), AB Sciex 5600 Triple TOF correction liquid (AB Science, USA), RevertAid™ First Strand cDNA Synthesis kit and Maxima™ SYBR-Green/Fluorescein qPCR Master mix (Thermo Fisher Scientific, Burlington, ON, Canada), and 3-(4,5-dimethylthiazol-2-yl)-5-(3-carboxymethoxyphenyl)-2-(4-sulfophenyl)-2H-tetrazolium) (MTS) kit (Promega, Wisconsin, U.S.A.). Apparatus used in this study were as follows: A Waters X BridgeTM BEH C18 analytical column XP (2.5 μm, 3.0 × 100 mm, Waters, Torrance, CA); AB 4000 Q-TRAP mass spectrum (ABSciex, USA); ACQUITYTM ultra-Performance LC (Waters, USA); Hemsr heometes (LBY-N7500A, China); Micro-plate reader (varioskan flash, USA); Ultra-pure Water Purifier Milli-Q (MILLIPORE, USA); Velocity 14 R high-speed refrigerated centrifuge (Dynamic, Australia); and MS2 basic mini shaker (IKA, Germany); rotary evaporator (IKA RV 10, IKA- Werke GmbH & Co. KG, Darmstadt, Germany).

### 2.2 Preparation of Spatholobus suberectus percolation extract

The extract of *S. suberectus* percolation (SSP) was prepared in accordance with previous literature with slight changes.^9^ Briefly, the dried stems of *S. suberectus* were blended in a blender and then extracted with 10 times the volume of 60% ethanol (v/w) using a percolating device. The filtrate was concentrated under reduced pressure using a rotary evaporator. The obtained percolation powder (20%) was then freeze-dried and stored at 4 °C until use.

### 2.3 Cell culture and treatment

BC cells, MDA-MB-231, and BT-549 cells (ATCC, Manassas, VA, USA) were used in this study. The cells were maintained in RPMI 1640 or glucose-containing (4.5 g/L) DMEM, supplemented with FBS (10% v/v), penicillin (100 U/ml), and streptomycin (100 μg/ml) in an atmosphere (5% CO_2_ at 37 °C). Cells (3 − 5 × 10^3^/well) were seeded onto 96-well plates and treated with various concentrations of *S. suberectus* (1.56–200 μg/ml). The growth inhibitory effect of *S. suberectus* on BC cells was determined using the MTS kit. The IC50 values of the drugs for the BC cell lines were measured using linear or nonlinear regression.

### 2.4 Cell viability assay

MTS assay was used to detect the IC50 of *S. suberectus* in BC cells. The cell suspension was then seeded into 96-well plates. After allowing the cells to adhere overnight, they were treated with different doses of *S. suberectus* for 48 h. At the endpoint, the medium of the cells was changed from containing *S. suberectus* to 20% MTS. The cells were incubated at 37 °C for 1.5 h. The optical density was measured at 490 nm using a microplate reader (Model 680, Bio-Rad). The data were plotted as the mean ± SD, and the IC_50_ was calculated using Prism 8.0 and n = 3.

### 2.5 Animals

6–7 weeks old female BALB/c nude mice were procured from Harlan Laboratories (Indianapolis, IN, USA) and maintained in a room with definite climatic conditions (22 ± 2 °C, 50 ± 10% relative humidity) with a 12 h light/12 h dark cycle and provided a standard diet with water ad libitum. The animals were housed in the Animal Unit of the University of Hong Kong. All experiments were approved by the Guidelines for Laboratory Animal Care and *Committee on the Use of Live Animals in Teaching and Research* (CULATR No: 4484–17). The xenograft assay was performed according to a previous study with minor changes.^36^^,^^37^ Briefly, 2 × 10^6^ MDA-MB-231 cells were implanted subcutaneously into the bilateral flanks of mice. Palpable and quantifiable tumors initially appeared 10 days after BC cell injection. The mice were randomly divided into three groups of six animals each: the vehicle control group, *S. suberectus* -L, and *S. suberectus* - H which received 0.4 and 0.8 g/kg/p.o. Daily respectively. The entire administration lasted 21 days. The tumor size and body weight of the animals were monitored every three days. At the endpoint, the mice were anesthetized with chloral hydrate, blood was obtained from the aorta, and plasma and serum were prepared immediately for metabolomic analysis. The tumor, heart, spleen, lung, liver, pancreas, and kidneys were collected in 4% PFA and liquid nitrogen for further analysis. The size of the tumor was determined using the calculation: 0.5 × length × width.^2^

After fixing, the tissues were dehydrated and embedded in molten paraffin. The samples were cut into small pieces. The sections were set on a microscope slide and the wax was removed using a solvent. After rehydration, the tissues were stained with hematoxylin and eosin.

### 2.6 Western blot analysis

Western blot assay was performed as previously described.^22^ BC cells (2 × 10^5^/well) were seeded in 6-well plates and added 25–100 μg/ml *S. suberectus* for 24 h. RIPA buffer contained protease/phosphatase inhibitor cocktail to extract the cells’ proteins. The protein was quantified using bicinchoninic acid and copper (II) sulfate solutions. Proteins were separated using 10% SDS-PAGE and transferred to a polyvinylidene fluoride (PVDF) membrane. After blocking with BSA (5%), membranes containing target protein were incubated with primary antibodies overnight at 4 °C. The primary antibodies are K-Ras (1:1000), Raf (1:1000), AMPK (1:1000), *p*-AMPK (1:1000), MEK (1:1000), *p*-MEK (1:1000), Erk1/2 (1:1000), *p*-Erk1/2 (1: 500), ACC (1:1000), FAS (1: 1000), EGFR (1:1000), and GAPDH (1:2000). Membranes were incubated with secondary antibodies after washing thrice with TBST at 25 °C. Mouse and rabbit antibodies were used as secondary antibodies. A chemiluminescence system was employed to visualize the protein bands. The target protein was detected by measuring the signal after membrane incubation with the substrate, suing the Gel-Doc system. The results were analyzed using Image Lab software.

### 2.7 Proteomic analysis

Proteomic analysis was performed to determine the differences in protein expression between the tumor tissues of mice. These mice were treated with 0.8 g/kg/d *S. suberectus* for 21 days in a xenograft model. Proteomic experiments were conducted by the BGI Genomics Co. Ltd. Software (Shenzhen, China). iTRAQ technology was used to identify and quantify proteins in the samples. The main procedures of the iTRAQ quantitative proteomics experiment are shown in Fig. 2A including protein extraction, quality control, proteolysis, peptide labeling, peptide separation by HPLC, and identification of peptides by mass spectrometry.

### 2.8 Metabolomic analysis

Briefly, 100 μL serum was added to 100 μL PBS containing 10 mM NEM, mixed with 1000 μL MeOH, and cultivated at −20 °C. Centrifugal at a speed of 13500×*g* for 15 min at 4 °C. The supernatant liquid was transferred into auto-sampler plastic vials and dried with nitrogen. The serum of all samples was stored at −20 °C for further use.

### 2.9 Metabolic profiling

The following techniques were used to conduct LC-MS/MS (Thermo Scientific, MA, U.S.A.) metabolomics analyses. A 25-min gradient program was combined with a linear gradient mobile phase made up of formic acid water (solvent A, 0.1%) and methanol (solvent B): 10.0–14.0 min (3–50% B); 14.0–18.0 min (50–95% B); 18.0–22.0 min (95-0% B); 0–3.0 min (0%–1% B); 3.0–10.0 min (1–3% B); 10.0–14.0 min (3–50% B). Multiple Reaction Monitoring (MRM) detection modes were applied for both positive and negative ion detection with an electrospray source (ESI). Using *m*/*z* 171.1⟶125.2, The internal standard Phe-d5 was measured. The flow rate was 0.6 ml/min, and the injection volume was 10 ml. An Ion spray voltage of 4500 V, curtain gas at 20 pressure, source temperature of 450 °C, ion source gas 1 at 40 psi, and ion source gas 2 at 40 psi were all part of the specific MS condition. AB Analyst Software gathered the UPLC-MS data (Version 1.6.2).

### 2.10 Targeted metabolic studies

The plasma concentrations of Try, Kyn, 5-HTP, and 5-HT were calibrated using common reference compounds and the targeted metabolic technique.^38^ Phe-D5 internal standard stock solution and standard stock solution (1 mg/ml) were prepared and kept at −40 °C. The UPLC-MS analysis was performed as previously described. By adding 6 g/100 ml charcoal activated powder to mouse plasma that had been cleared of endogenous components, blank plasma was made for quality control. After adding Try, Kyn, 5-HTP, and 5-HT, and standard solutions to the blank, QC samples were created with three different concentrations and processed in the same manner as described previously. The QC samples were used to create standard curves with eight different concentrations, which were then estimated using 1/X weighted least-squares regression. Succession of QC samples and calibration curves was performed at eight sample intervals.

### 2.11 RT-PCR analysis

Trizol reagent was used to extract total RNA from mouse tumor tissues according to manufacturer's instructions. Using a First Strand cDNA Synthesis Kit, 3-g of total RNA was reverse-transcribed into cDNA for 1 h at 42 °C. With 1 μl of cDNA, 12.5 μl of MaximaTM SYBR Green/Fluorescein qPCR Master Mix (2X), 1 μM forward primer, and 1 μM reverse primer in a final volume of 25 μl, real-time PCR was carried out using an IQTM5 real-time PCR detection system (Bio-Rad, USA). With the use of particular primers, the cDNA was amplified in 45 cycles at 94 °C for 30 s, 55 °C for 30 s of annealing, and then 72 °C for 50 s, with a final incubation at 72 °C for 7 min. Primers employed for RT- PCR are outlined in Supplementary Table 1.

### 2.12 Determination of liver function tests in the serum of mice

The serum of the mice was used to determine liver functional indices, including creatinine (CREA), blood urea nitrogen (BUN), alanine transaminase (ALT), and aspartate transaminase (AST). The detection kits were procured from the Nanjing Jiancheng Bioengineering Institute, Nanjing, China.

### 2.13 Statistical analysis

The collected MS data were examined for metabolomics using the MarkerView1.2 program (AB Sciex, USA). The range of data measurements was 0.5–30 min. This range had with a minimum peak intensity of 10% of the base peak and a maximum peak width of 25 ppm. Six peaks with the same retention duration were detected; the mass-to-charge ratio difference was 20 ppm and the retention time variation was 0.05 min. To give all variables in this study equal weights regardless of their magnitude, Pareto scaling was employed to diminish the significance of the intensity. After preprocessing, SIMCA-P software 11.5 was used to carry out partial least square discriminant analysis (PLS-DA), a well-known and supervised method for multivariate statistical analysis that has been regularly used in metabolomic research (Umetrics, Umea, Sweden). To ensure the quality of the multivariate models and prevent the risk of overfitting, model parameters, such as R2Y and Q2Y, were examined. The parameter known as “variable importance in the projection” (VIP) measures the relevance of each variable in a PLS-DA model and summarizes the importance of the variables in both the X and Y models. In the tested model, a VIP score variable close to or larger than one can be deemed significant. The p-value of the metabolites was determined using the Student's t-test in addition to the VIP value. To identify potential biomarkers, variables with VIP >1 and p < 0.05, were chosen for additional SPSS 20.0 statistical analysis. The relative amounts of different metabolites were represented on a heat map using HemI 1.0 software tools, and their hierarchical clusters were examined. ChemSpider Service in PeakView (AB Science) carried out metabolite identification, whereas PubChem (https://pubchem.ncbi.nlm.nih.gov/) and HMDB (http://www.hmdb.ca/) were used to search for metabolite information. Statistical differences in metabolites from quantitative MS spectra across groups were determined using a one-way analysis of variance (ANOVA). Statistics significance was set at p < 0.05.

## 3 Results

### 3.1 S. suberectus inhibits TNBC *in vitro* and *in vivo*

**Fig. 1:**
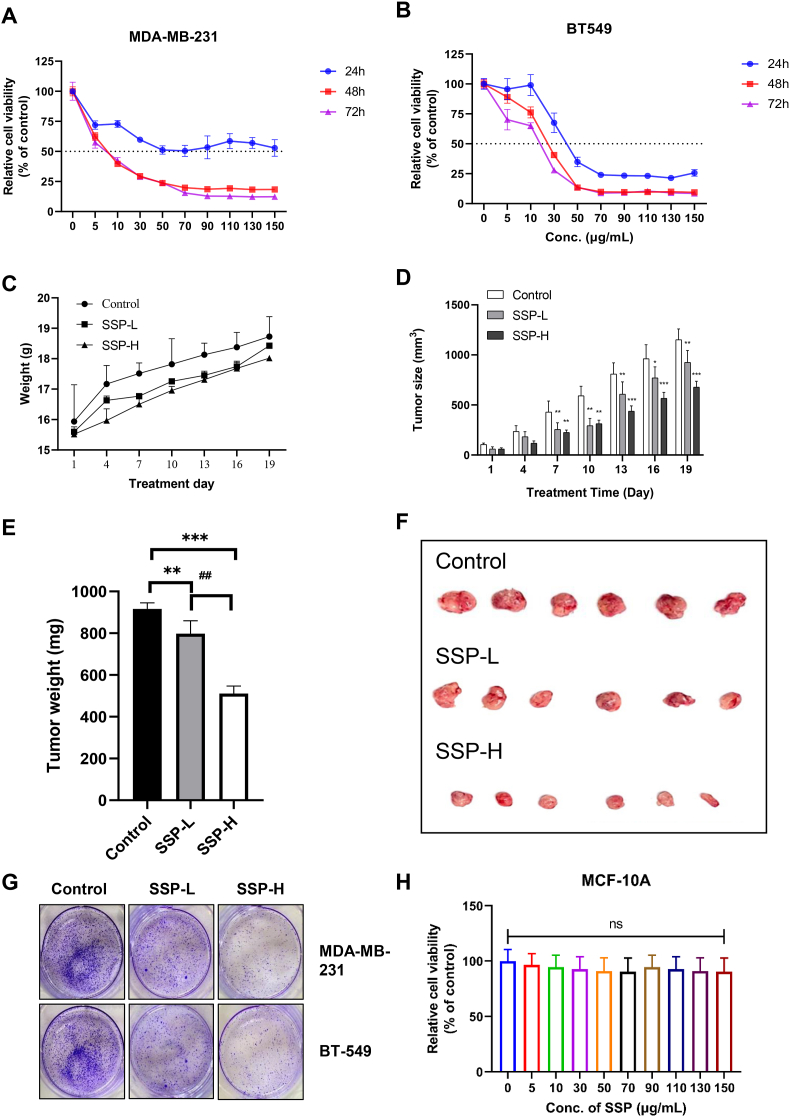
Inhibitory roles of SSP on TNBC *in vitro* and *in vivo*. **(A and B)** Representative pictures of cell viability assay of BC cells after treatment of different concentrations of SSP **(C)** Representative pictures of mice xenografts treated with low and high doses of SSP for 19 days analyzed the bodyweight curve **(D)** tumor size **(E)** tumor weight **(F)** excised tumor from the mice **(G)** Representative pictures of colony formation assay: the anti-clonogenic ability of SSP against MDA-MB-231 and BT549 were examined. **(H)** Various concentrations of *S. suberectus* do not affect non-TNBC cell lines, MCF-10A. The data were analyzed by mean ± SD with one-way ANOVA. ∗P < 0.05, ∗∗P < 0.01, ∗∗∗P < 0.001, ns, non-significant.

The relative cell viability of *S. suberectus* (0–150 μg/ml) was confirmed in BC cell lines for 24–72 h *in vitro* (Fig. 1A and B). Different concentrations of *S. suberectus* showed a noteworthy inhibitory effect on TNBC cells in time and dose-dependent manners. According to the increasing dosage observed in MDA-MB-231 and BT-549 cancer cell lines, *S. suberectus* exhibited notable growth-inhibitory effects. We used the MDA-MB 231 xenograft model to evaluate the anti-TNBC effect of *S. suberectus in vivo*. Mice were administered orally with *S. suberectus* -L (0.4 g/kg b. w) and *S. suberectus* –H (0.8 g/kg b. w) for 19 days, and the tumor size and the weight of the mice were measured every three days. The results showed a noteworthy difference in body weight, size of the tumor, and tumor weight between the control and *S. suberectus* treated groups (Fig. 1C–1F). *S. suberectus* –H exerted significant tumor growth inhibition based on the inhibition of tumor weight and size compared to the control group. Fig. 1G shows that *S. suberectus* –H exerted a potent anti-clonogenic effect compared to the control and low doses of *S. suberectus* against both BC cell lines. Fig. 1H demonstrates that various concentrations of *S. suberectus* do not affect non-TNBC cell lines, MCF-10A. Thus, SSP specifically inhibits TNBC cell growth without affecting non-TNBC cells, suggesting that it may have a more favorable safety profile than other anticancer drugs. Thus, *S. suberectus* inhibits TNBC cell growth in *in*-*vitro* and *in vivo*.

**Fig. 2:**
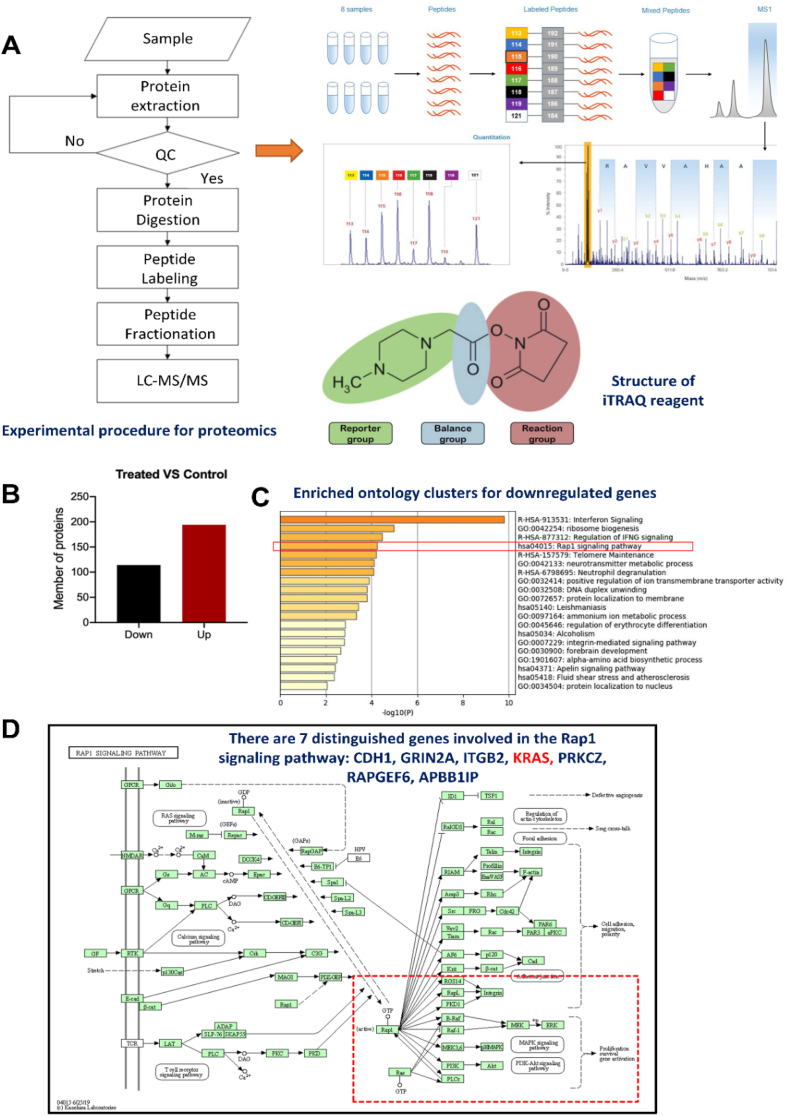
Explore the study of SSP inhibits BC via downregulating the K-Ras-Raf signaling pathway by proteomics. (A) Flowchart of the experiment (B) Up-down regulated proteins involved in the BC progression (C) Enriched ontology clusters for downregulated genes (D) distinguished genes involved in Rap1 signaling pathways.

### 3.2 S. suberectus inhibits TNBC via downregulating the K-Ras-Raf signaling pathway by proteomic analysis

As iTRAQ technology can be used for almost any protein sample, it can quantitatively compare up to eight samples in one experiment. Thus, we used iTRAQ technology to identify differential proteins in the tumor tissues of the *S. suberectus* treatment and control groups. A flowchart of the experiment is shown in Fig. 2A.

**Fig. 3:**
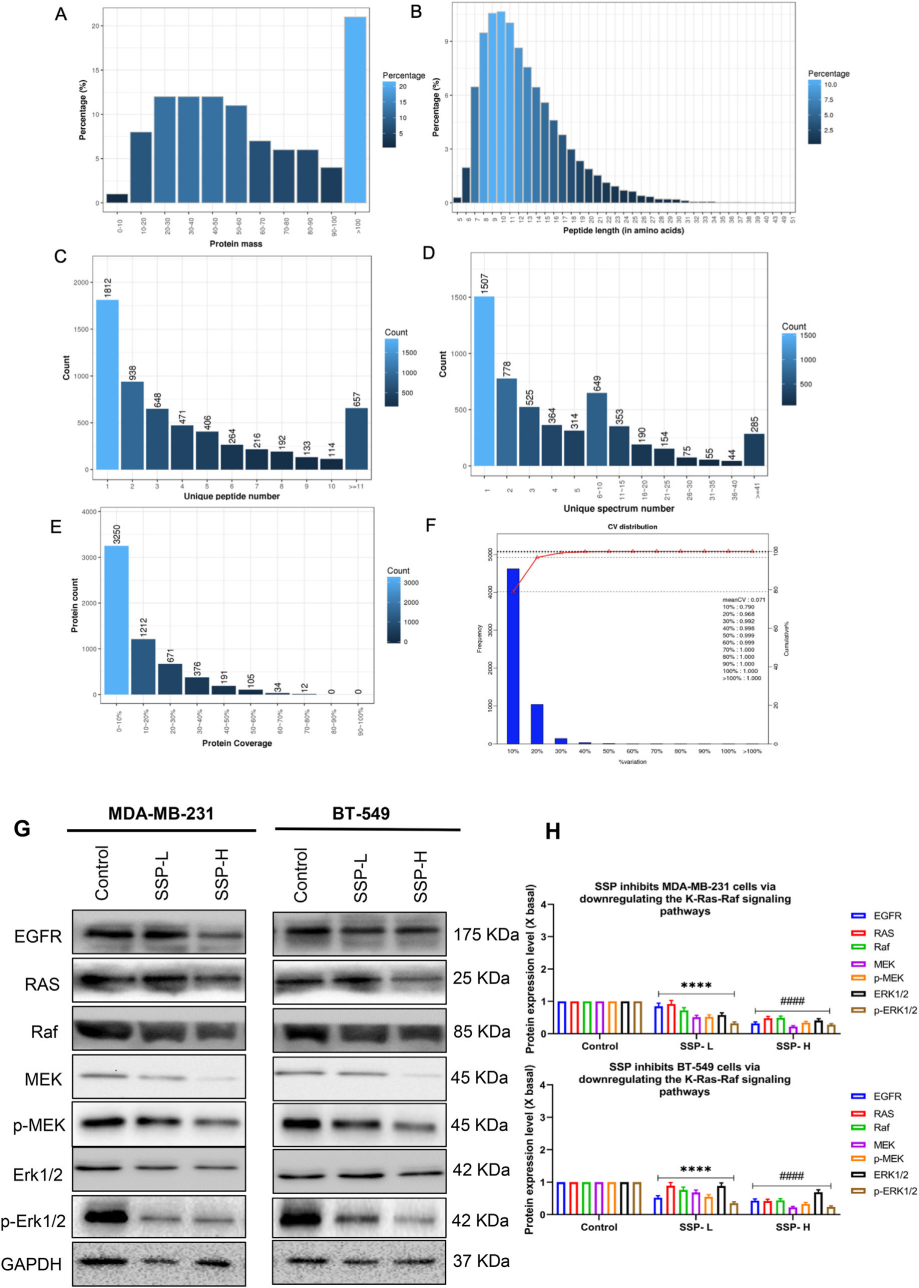
Identification of the distinguished proteins in tumor tissues of the control and SSP treated group. The attributes of identified proteins include (A) protein mass, (B) peptide length, (C) unique peptide number, (D) unique spectra number, (E) area coverage of proteins, and (F) percentage of variation. (G) SSP inhibits tumor development through downstream regulation of the KRas-Raf signaling in TNBC cells and xenograft mice models. Protein expression on MDA-MB-231 and BT549 after SSP-L and SSP-H treatment, as determined by western blotting. (H) The quantification of the target protein was calculated with a densitometer. The values are expressed as the fold of change (X basal), in Mean ± SEM, where n = 3. ∗∗∗∗p < 0.0001 was considered a significant result compared to control; ####p < 0.0001 was considered a significant result compared to SSP-L.

During the iTRAQ investigation, 393,315 spectra were generated. False discovery rate (FDR) can effectively control false-positive results. As a result, it can maximize the number of differentially expressed proteins that can be used as a crucial indicator. Under the “1% FDR” filtering standard, a total of 30,422 peptides and 5851 proteins were found. The identified proteins contained at least one unique peptide. The attributes of the identified proteins included protein mass distribution, peptide length distribution, distribution of unique peptide number of proteins, distribution of unique spectra number of proteins, and distribution of identification area coverage of proteins, as shown in Fig. 3A–E. Coefficient of variation (CV) was used to evaluate the repeatability of protein identification. The CV is defined as the ratio of the standard deviation to the average value. SD is the standard deviation of the presence of a particular protein among different measurements. Mean is the average probability of a specific protein from different measurements. The lower the CV, the better is the reproducibility. Based on the quantification repeat analysis, the average CV was 7.1%, and 96.8% of the proteins had CVs below 20% (Fig. 3F). This indicated that the results of the identified proteins were reliable.

ITRAQ quantification was performed using IQuant software. In this study, we compared the differentially expressed proteins in the treated and control groups. In a single experiment, proteins with fold change >1.2 and Q-value of 0.05, were tested for significant differences. The resulting differential protein was tested for repeated experimental data with a fold change >1.2 (average ratio of the comparison group) and a P-value of 0.05. (*t*-test for the comparison group). After screening, 194 proteins were upregulated and 114 proteins were downregulated. In total, 308 genes were identified (Fig. 2B). We threw 114 downregulated genes into the signaling pathway by Metascape analysis to clarify the regulation of *S. suberectus* signaling pathways. The results indicated that the top five signaling pathways that interfered with *S. suberectus* were the interferon signaling pathway, ribosome biogenesis pathway, regulation of the IFNG signaling pathway, Rap 1 signaling pathway, and telomere Maintenance pathway (Fig. 2C). Among the interfering signaling pathways, Rap1 Signaling Pathway is closely linked to cell proliferation. The Rap1 Signaling pathway includes seven distinct genes: CDH1, GRIN2A, ITGB2, KRAS, PRKCZ, RAPGEF6, and APBB1IP (Fig. 2D).

Proteinomics is a powerful tool for identification of disease biomarkers and drug targets. The Rap1 signaling pathway was significantly regulated by *S. suberectus* as there were seven different genes involved in this pathway. The Rap1 signaling pathway includes K-Ras, an oncogene that participates in cell growth. Ras is a GTP binding protein and is also the target for cancer drugs.^39^ KRAS is the most frequently mutated RAS subtype in BC closely associated with poor prognosis.^40^ The triggering of the RAS/MAPK pathway promotes immune outflow in TNBC.^41^ The phosphorylation of ERK is elevated in TNBC, implying the initiation of the Ras signaling pathway.^42^^,^^43^ Proteomics analysis showed that *S. suberectus* significantly downregulated the K-Ras gene in tumor tissues. We found that K-Ras and its downstream proteins were downregulated by *S. suberectus* (Fig. 3G) in both MDA-MB-231 and BT-549 cells. The intensity of K-Ras and its downstream proteins were downregulated in MDA-MB-231 and BT549 after *S. suberectus* –H and *S. suberectus* -L treatment when compared to the control (Fig. 3H). The results suggest that *S. suberectus* may suppress the K-Ras-Raf signaling pathway to inhibit cell proliferation.

### 3.3 S. suberectus inhibits tumor development through the downstream signaling pathway of K-Ras-Raf in TNBC cells and xenograft mice models

**Fig. 4:**
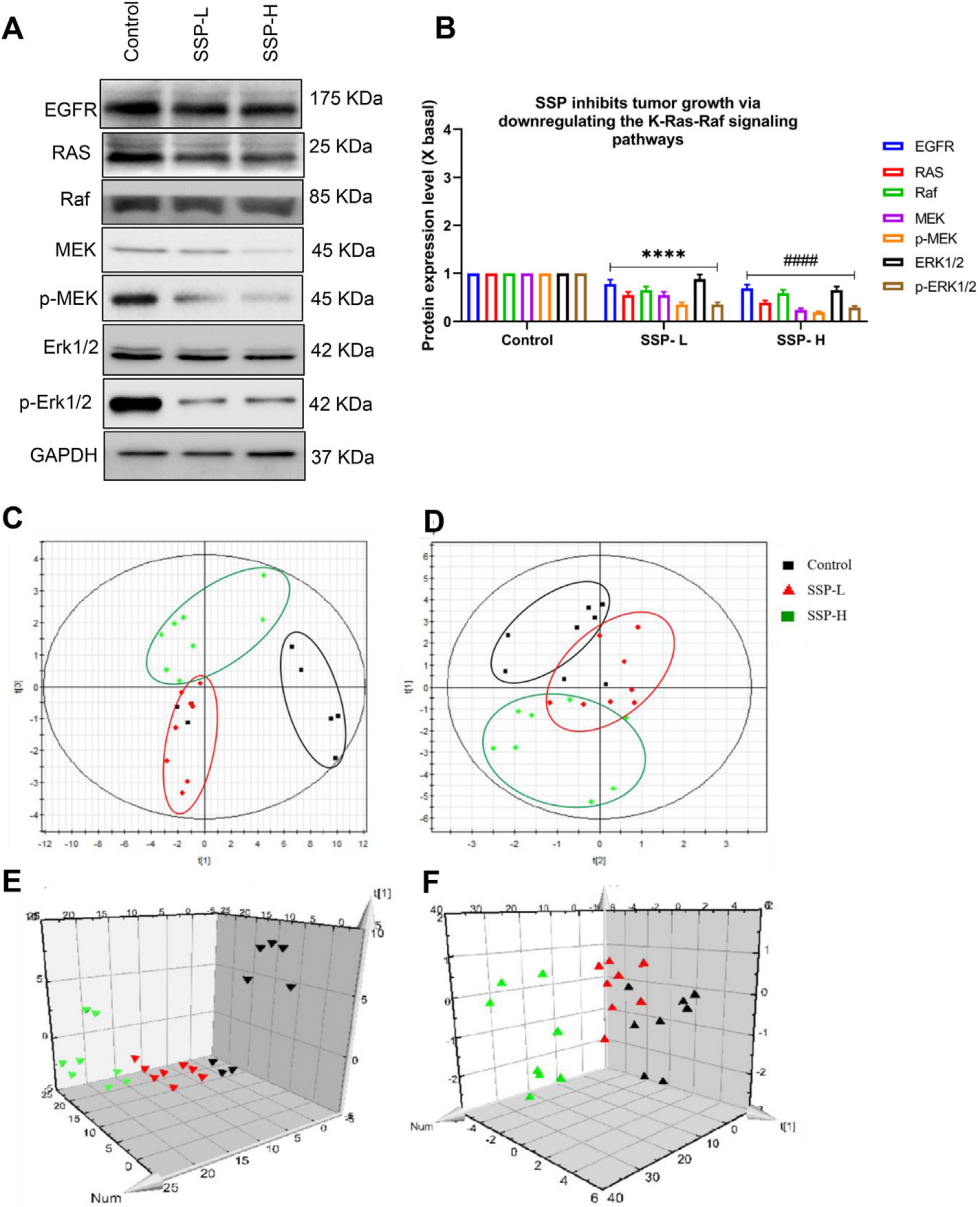
(A) Protein expression on tumor tissues of xenografted mice after SSP-L and SSP-H treatment, as determined by western blotting (B) The quantification of the target protein was calculated with a densitometer. The values are expressed as the fold of change (X basal), in Mean ± SEM, where n = 3. ∗∗∗∗p < 0.0001 was considered a significant result compared to control; ####p < 0.0001 was considered a significant result compared to SSP-L. PLS-DA score plots containing scatter plots. Each group was distinguished by a color circle and corresponding color. Black represents the normal group, and red and green represent the low and high-dose groups of SSP. (C, E) Positive score plots: R2X = 0.58, R2Y (cum) = 0.703 and Q2 (cum) = 0.327. (D, F) Negative ionization modes: R2X = 0.473, R2Y (cum) = 0.439 and Q2 (cum) = 0.362.

Based on the outcome of ITRAQ quantification, we explored the downstream pathways of KRas-Raf signaling in TNBC cells and xenograft mouse models. Western blotting was performed to elucidate the molecular mechanisms of *S. suberectus* in TNBC suppression. Treatment of MDA-MB-231 and BT549 (Fig. 3G) cells downregulated the expression of EGFR, Ras, Raf, MEK, *p*-MEK, ERK1/2 and *p*-ERK1/2. This result was consistent with the treatment of *S. suberectus* –H and *S. suberectus* -L on the tumor tissues of xenografted mice which downregulated the expression of EGFR, Ras, Raf, MEK, *p*-MEK, ERK1/2 and *p*-ERK1/2 (Fig. 4A). Similarly, The intensity of K-Ras and its downstream proteins were downregulated in tumor tissues after *S. suberectus* –H and *S. suberectus* -L treatment when compared to the control (Fig. 4B). Thus, these results show that *S. suberectus* significantly inhibits TNBC cell growth through the downregulation of the KRas-Raf signaling pathway *in vitro* and *in vivo*.

### 3.4 S. suberectus influences serum metabolites analyzed by metabolomics

SIMCA-P software 11.5 was used to input all the data, which included retention duration, peak intensity, and exact mass. Fig. 4 (C–F) displays the positive and negative ion modes of PLS-DA for the control, *S. suberectus* –H, and *S. suberectus* -L groups. This mathematical model was developed, and 7-fold cross-validation was performed before PLS-DA was modified to most effectively assess the difference. Fig. 4 (C–F) displays the results of PLS-DA with positive (A) and negative (B) ionization modes. The difference between R2Y (cum) and Q2 (cum) was 0.3 after fitting the data, which had R2X = 0.580, R2Y = 0.703, and Q2 (cum) = 0.327 in positive ionization modes R2X = 0.473, R2Y (cum) = 0.439, and Q2 (cum) = 0.362 in negative ionization modes. This shows that the nobly observed group separation was attributed to each of the well-distinguished groups. In general, the PLS-DA score showed that there was a collection among *S. suberectus* treatment groups in the negative ionization type, while there was a small overlap between the *S. suberectus*-H group, and the control, clearly showing that there were notable markers that were able to change after treatment. Furthermore, there was an area where the treatment groups overlapped in both the positive and negative modes. The results indicated that metabolic profiles differed significantly between different doses based on mutual comparisons, and that these metabolic profiles could be used as biomarkers to determine groups.

### 3.5 S. suberectus promotes fatty acid oxidation markers by metabolomics analysis

Supplementary Table 2 shows that 10 distinct metabolites were detected in the control and *S. suberectus* treated groups based on the settings for scanning potential metabolites. These metabolites were classified as follows: lipid and FA metabolism (LysoPC (16:0) and LysoPC (18:0)), FA beta-oxidation (N-phenyl acetyl glycine and acetylcarnitine), citrate cycle (AA, FMA, and CA), butanoate metabolism (3-hydroxybutyric), bile acid metabolism (GDCA) and d-glutamine metabolism (glutamic acid). In general, this alteration in the metabolic profile indicates that *S. suberectus* may be involved in the lipid metabolic pathway.

**Fig. 5:**
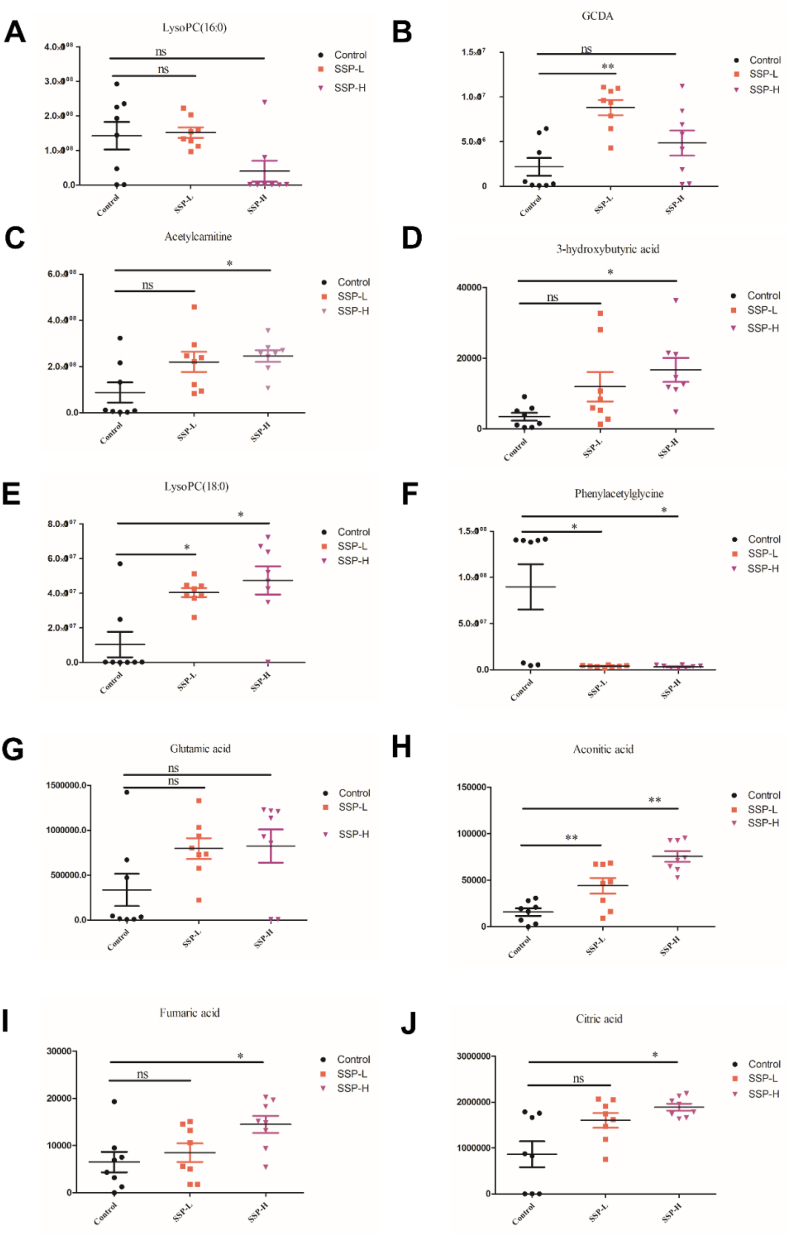
SSP promotes fatty acid metabolites. Metabolomics data were misrepresented by the mean ± SD of the scatter distribution. Each group was distinguished by color. Black represented the normal group; red and orange represented the high and low-dose groups of SSP. ∗p < 0.05; ∗∗p < 0.01.

Compared with the control, the listed metabolites in the treated groups were considerably altered (P < 0.05/P < 0.01) (Fig. 5). Most metabolites, including LysoPC(16:0), LysoPC(18:0), GDCA, acetylcarnitine, glutamic, 3-hydroxybutyric, AA, FMA, and CA were significantly elevated (p < 0.05/p < 0.01), when N-phenyl acetyl glycine was considerably decreased (p < 0.05). The constitutes of LysoPC (18:0), GCDA, and AA in the *S. suberectus* –H group were much more abundant than those in the *S. suberectus* -L group (p < 0.05).

**Fig. 6:**
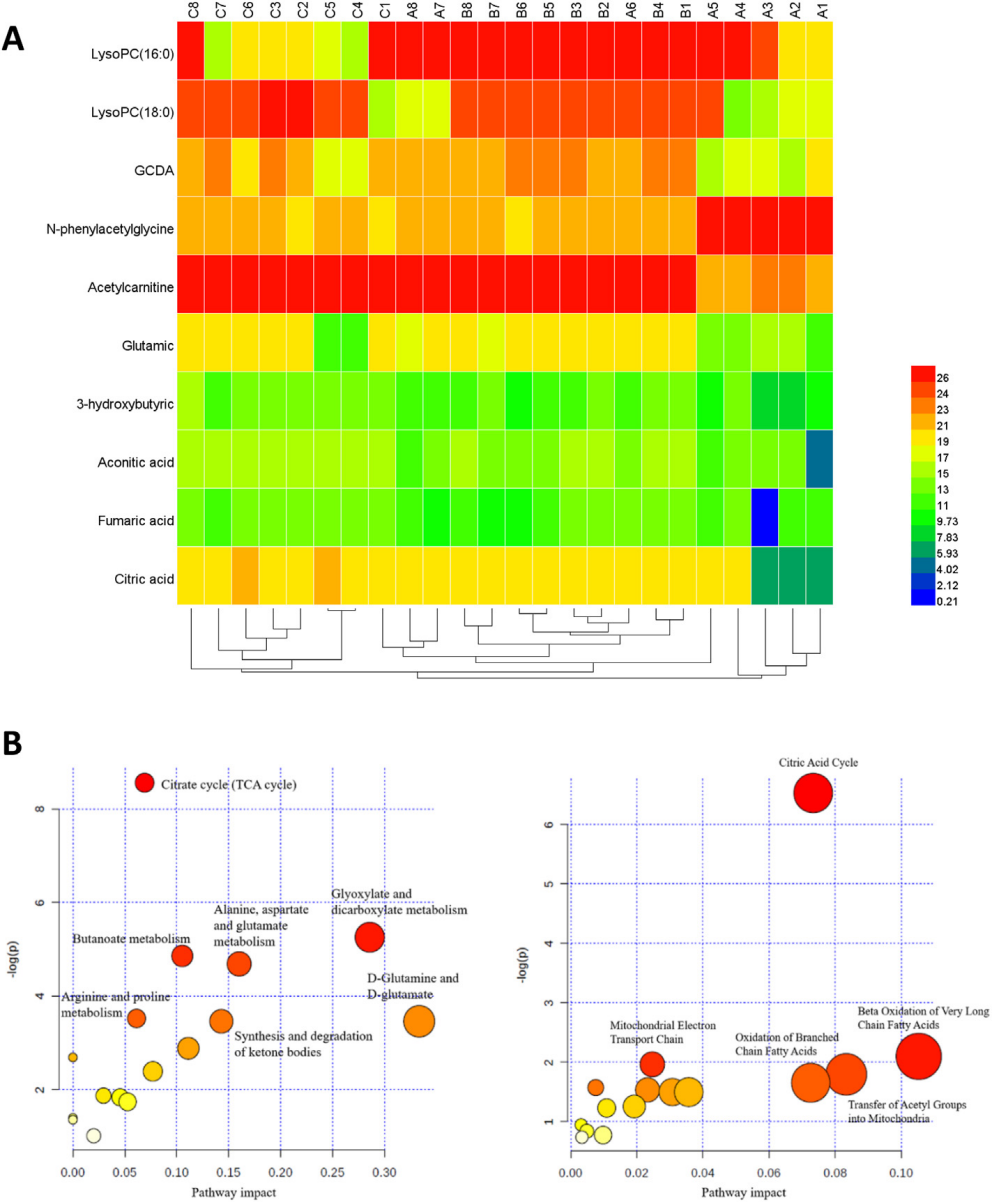
Thermograph and Cluster Analysis. (A) On the left, each potential biomarker is arranged vertically. On the top, the samples of each group are arranged horizontally, and on the bottom, the cluster analysis of all samples is performed (Control: A1∼A8; SSP-L: B1∼B8; SSP-H: C1∼C8; SSP). (B) SSP alters the metabolites in the tumor tissues analyzed by KEGG and SMPDB. The abscissa represents the influence factors of each pathway, and the ordinate represents the significance of the change in the pathway. (Left. Pathway analysis from KEGG; Right. Pathway analysis from SMPDB).

Fig. 6A shows the inductive analysis using a heat map and clustering analysis. In this study, Euclidean distance was employed to mathematically define the similarity between two things. After these data were processed, the square represented metabolites as a heat map. A low response to a high is depicted by a gradual change in the hue from blue to red, which represents their levels. Given statistical alteration, the change of metabolites between control and *S. suberectus* treated groups in the heatmap was consistent with the depiction of data in a table. From the perspective of the cluster, it was evident that each group could differ from the others in general. This suggest that the alteration of metabolites could be used to classify the groups according to whether they were affected by *S. suberectus*. However, four different individuals, two from the control group (A7 and A8) that clustered with *S. suberectus* -L, and another two from the *S. suberectus* –H group (C4 and C5) that clustered with the control group, were interestingly clustered due to similar changes in several metabolites, including LysoPC (16:0), LysoPC (18:0), GDCA, 3-hydroxybutyric, FMA, and CA.

The pathway analysis result based on KEGG and SMPDB are shown (Fig. 6B). In enrichment analysis, the p-value can distinguish the pathways in which metabolites are altered significantly, and the impact shows the priority of each pathway. Both databases share the same consensus, suggesting that *S. suberectus* interferes with energy metabolism. Based on these two analyses, the TCA pathway, which includes AA, FA, and CA, is considered to be one of the most obvious pathways in which metabolites are altered. Comprehensively, the KEGG pathway recognized glutamine, glyoxylate, ketone bodies, alanine, aspartate, and glutamate metabolism as the predominant impact among these pathways, while SMPDB thought that FA beta-oxidation played a major role in these metabolic pathways. Therefore, *S. suberectus* may exert its regulatory effects via FA oxidation.

### 3.6 S. suberectus prevents lipogenesis and promotes fatty acid oxidation in the tumor tissues by qPCR analysis

**Fig. 7:**
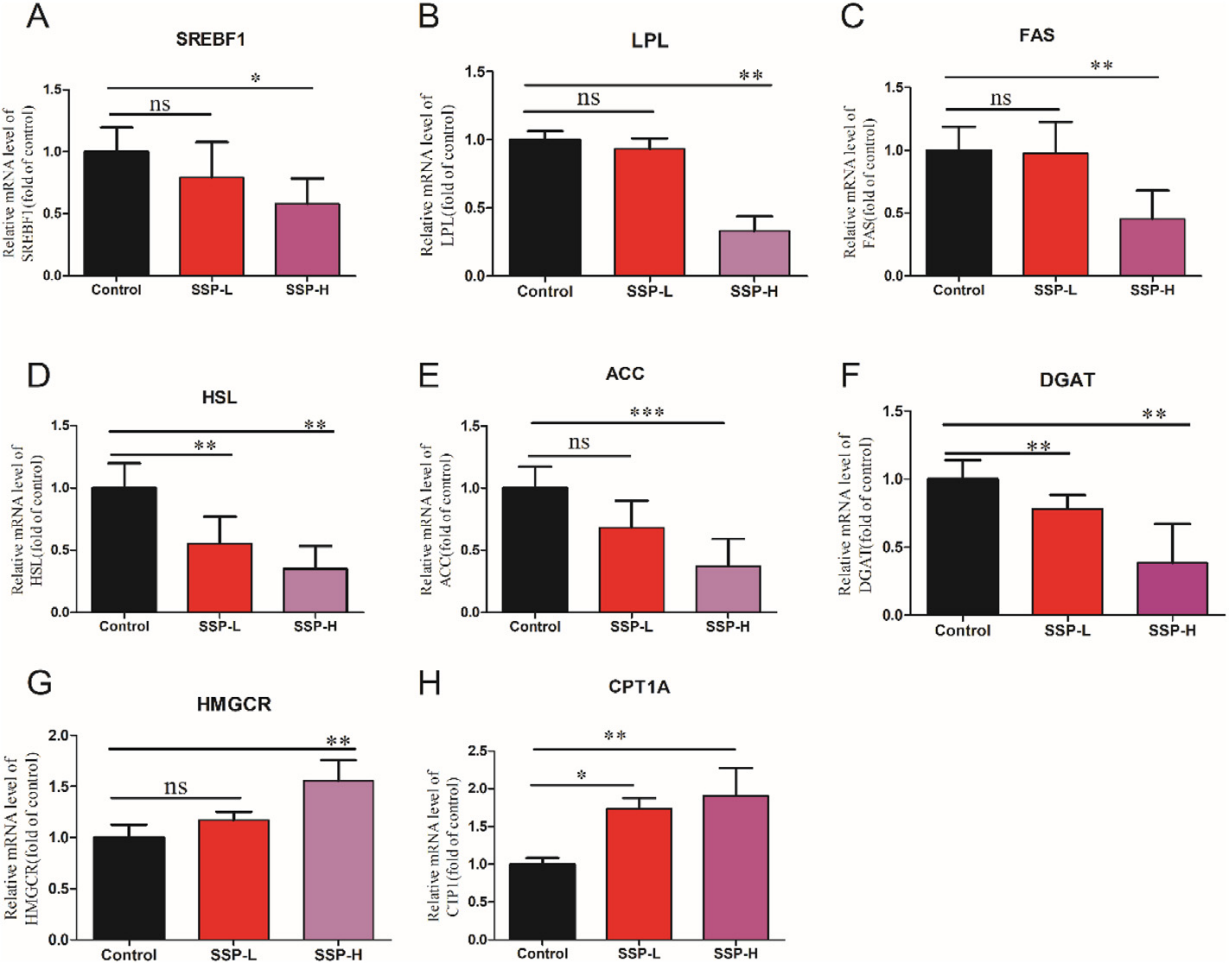
Influence of SSP on fatty acid markers in the tumor tissues of the mice. SSP treatment significantly prevented lipogenic markers (A–F) and promoted fatty acid oxidation markers (G–H) in the tumor tissues, which were analyzed by qPCR. ∗p < 0.05 ∗∗p < 0.01 indicated that there were significant differences in the control and SSP treated groups.

As seen in FA metabolism, there was a significant downregulation in SREBF1, FAS, ACC, DGAT, LPL, and HSL in comparison to the control (p < 0.05 or p < 0.01), and gene expression of FAS, LPL, and DGAT was substantially reduced in the *S. suberectus* –H group compared to the *S. suberectus* -L group (p < 0.05, p < 0.01) (Fig. 7). HMGCR and CPT1 gene expression in *S. suberectus* –H was remarkably upregulated (p < 0.01) compared to that in the control. *S. suberectus* inhibited endogenous FA synthesis, and promoted HMGCR-mediated actions, and its effect on FAS, LPL, DGAT, CPT1, and HMGCR became more apparent at higher doses.

### 3.7 S. suberectus prevents lipogenesis in the mice tumor tissues by Western blot

**Fig. 8:**
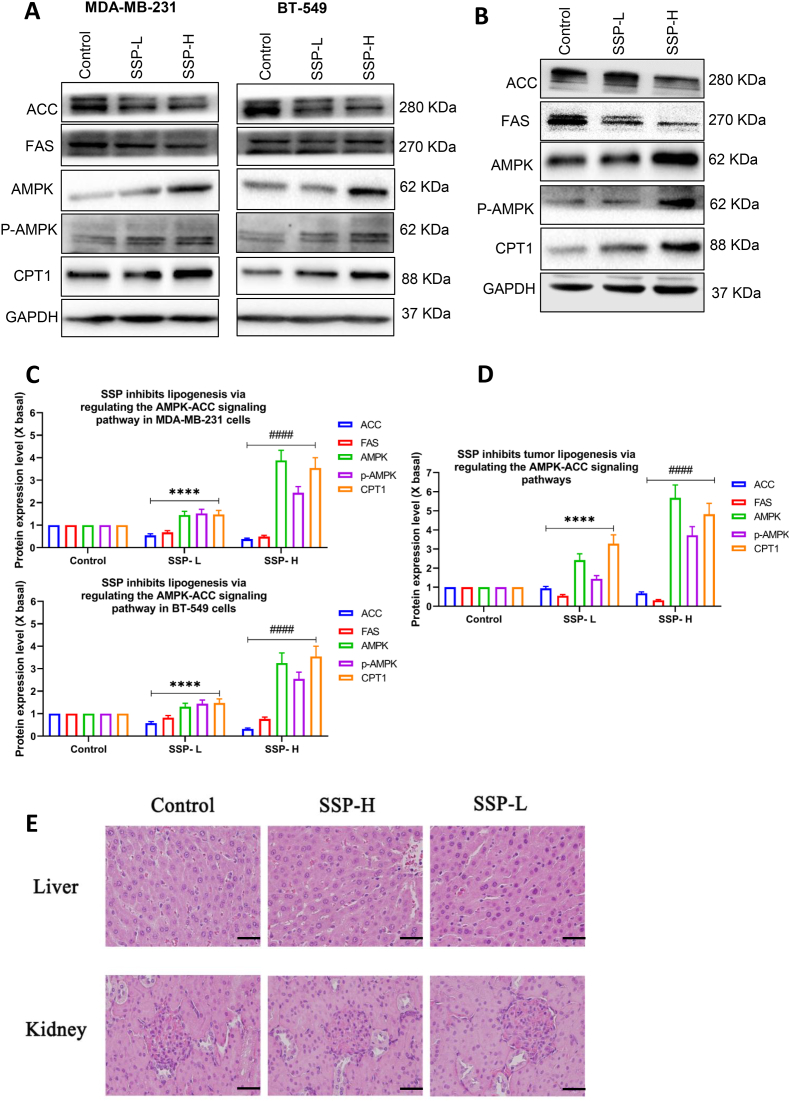
SSP prevents lipogenesis through downstream and upstream pathways in TNBC cells and xenograft mice models. (A) Lipogenic marker expression on MDA-MB-231 and BT549 after SSP-L and SSP-H treatment, as determined by western blotting. (B) Lipogenic marker expression on tumor tissues of xenografted mice after SSP-L and SSP-H treatment, as determined by western blotting (C) The quantification of the lipogenic marker expression on MDA-MB-231 and BT549 after SSP-L and SSP-H treatment, which was calculated with a densitometer. The values are expressed as the fold of change (X basal), in Mean ± SEM, where n = 3. ∗∗∗∗p < 0.0001 was considered a significant result compared to control; ####p < 0.0001 was considered a significant result compared to SSP-L. (D) The quantification of the lipogenic marker expression on tumor tissues of xenografted mice after SSP-L and SSP-H treatment, which was calculated with a densitometer. The values are expressed as the fold of change (X basal), in Mean ± SEM, where n = 3. ∗∗∗∗p < 0.0001 was considered a significant result compared to control; ####p < 0.0001 was considered a significant result compared to SSP-L. (E) Histopathological observation of liver and kidney in mice. Each column from left to right represents the normal group and the high and low dose of SSP groups respectively. The upper and lower rows represent the respective liver and kidney tissues of mice (scale bar = 40 μm, magnification ×400).

Based on metabolomics and thermocluster analysis, we explored lipogenesis markers in TNBC cells and xenograft mouse models. Western blot assay was performed to elucidate the molecular mechanisms of *S. suberectus* on lipogenic markers in mouse tissues and thereby TNBC suppression. Treatment of MDA-MB-231 and BT549 (Fig. 8A) cells with *S. suberectus* –H and *S. suberectus* -L downregulated the lipogenic marker expression of ACC, FAS, AMPK, CPT1 and *p*-AMPK. This result was consistent with the treatment of *S. suberectus* –H and *S. suberectus* -L in the tumor tissues of xenograft mice which downregulated the expression of ACC, FAS, AMPK, CPT1 and *p*-AMPK (Fig. 8B). The intensity of lipogenic marker expression in MDA-MB-231 and BT549 was significantly decreased after *S. suberectus* –H and *S. suberectus* -L treatment compared to the control (Fig. 8C). Similarly, the intensity of lipogenic marker expression in tumor tissues was also significantly reduced after *S. suberectus* –H and *S. suberectus* -L treatment compared to the control (Fig. 8D). These results show that *S. suberectus* significantly inhibits lipogenic markers *in vitro* and *in vivo*.

### 3.8 Safety assessment of S. suberectus on the liver and kidney

Compared with the control group, there were no obvious differences in CREA, BUN, AST, and ALT in the *S. suberectus* treatment groups. This suggest that *S. suberectus* might not damage kidney and liver functions (Supplementary Table 3). The hepatocytes of the control group showed regular hexagons with similar-sized nuclei, and a rope-like supply and the bounds among individual chains were visible (Fig. 8E). Additionally, there was no detectable limit for symmetrically red-dyed air quality vesicles in the cytoplasm. A medial location was also chosen for the blue-stained nucleus. Hepatocytes that had been exposed to *S. suberectus* displayed a normal-sized nucleus as the control, a clear hepatic sinusoid, and a clear rope-like chain, demonstrating that *S. suberectus* has no negative effects on hepatocytes (Fig. 8E). HE staining of the kidney showed that the Malpighian corpuscle appeared as an ellipse with a clear boundary in the *S. suberectus* and control groups. Additionally, there were many kidney glomeruli and tissues in stable trials. These trials included the integrity of the nuclear structure, apparent structure, and no vacuolation, suggesting that *S. suberectus* had no adverse effects on the kidney tissue (Fig. 8E). Overall, Based on the findings, *S. suberectus* prevents TNBC by downregulating the K-Ras-Raf signaling pathway and inhibiting lipogenesis.

## 4 Discussion

BC is the most prevalent cancer worldwide.^1^ TNBC is a type of BC that is ER, PR, and HER2 being negative, and has a poor prognosis. Currently, there are no effective management strategies for TNBC. The current treatment for TNBC is mainly chemotherapeutic drugs, accompanied by noticeable side effects, including liver and kidney toxicity.^44^ Drug resistance is another significant problem in BC treatment. Chemotherapy reduces the effectiveness of anticancer drugs because cancer cells acquire DNA mutations, alter gene expression, slow proliferation, and decrease metabolism.^45^ Therefore, it is crucial to develop a more effective approach to combat TNBC.

As TCM has thousands of years of experience in treating tumors, it is promising to use this knowledge to find solutions to these problems. *S. suberectus* can theoretically be applied to the treatment of BC in TCM as it stimulates blood circulation and improves the microenvironment of the body. Our research team has focused on the use of *S. suberectus* for the treatment of BC for many years. Previously, we found that the aqueous extract of *S. suberectus* triggered apoptosis of BC cells *in vivo* and *in vitro*.^9^^,^^22^, ^23^, ^24^^,^^46^, ^47^, ^48^, ^49^, ^50^ The present study results were also consistent with earlier studies showing that *S. suberectus* exhibited a significant inhibitory effect and potent anti-clonogenic ability on TNBC cells in a time and dose-dependent manner. An animal model investigation also revealed that *S. suberectus* exerted significant tumor growth inhibition based on the inhibition of tumor weight and size in comparison with the control. An earlier study investigated the antiproliferative effect of *S. suberectus* vine stem extract on rat C6 glioma cells. It identified regulation of ROS, mitochondrial depolarization, and P21 protein expression as potential mechanisms of action resulting in a broad spectrum of anti-cancer properties.^51^

Proteomic technology was employed to elucidate the anti-TNBC mechanism of *S. suberectus*. The results showed that *S. suberectus* downregulated 114 differential genes in tumor tissues. Based on the Metascape analysis, we identified the top five downregulated genes in the signaling pathway that interfered with *S. suberectus*, which included the Interferon Signaling Pathway, Ribosome Biogenesis Pathway, Regulation of IFNG Signaling pathway, Rap 1 signaling pathway, and Telomere Maintenance pathway. Rap1 Signaling is associated with cell proliferation, and seven differentiated genes enriched in this pathway: CDH1, GRIN2A, ITGB2, KRAS, PRKCZ, RAPGEF6, and APBB1IP. *S. suberectus* alters the signaling pathway in TNBC cells. Our present study outcomes were consistent with an earlier investigation, which determined the mechanism of action of flavonoids and glycosides on signaling pathways based on the application of proteomic study using the SILAC method.^52^

Ras is an oncogene gene closely related to cancer development, thus Ras was found to be a well-recognized drug target.^53^ KRAS is a signaling protein that plays a critical role in the regulation of cell growth, differentiation, and survival.^54^ It is a member of the RAS family of small GTPases and is frequently mutated in human cancers.^55^ Recent studies have shown that KRAS signaling is closely linked to lipid metabolism.^56^^,^^57^ One of the key pathways regulated by KRAS is the PI3K/Akt/mTOR pathway, which plays a critical role in lipid metabolism and is frequently dysregulated in cancer.^58^ KRAS activation promotes lipid synthesis and storage by increasing the expression of key lipogenic enzymes such as FAS and ACC.^56^ In addition to promoting lipid synthesis, KRAS signaling also regulates lipid droplet formation and turnover. Lipid droplets are intracellular organelles that store neutral lipids such as triglycerides and cholesterol esters.^59^ Recent studies have shown that KRAS activation promotes lipid droplet formation by regulating the expression of key genes involved in lipid metabolism, such as perilipin and adipose differentiation-related protein.^60^ Several preclinical studies have shown that targeting key enzymes involved in lipid metabolism, such as FASN and ACC, can inhibit the growth of KRAS-driven tumors.^61^ The close link between KRAS signaling and lipid metabolism suggests that targeting lipid metabolism may be an effective strategy for the treatment of KRAS-driven cancers. We firstly demonstrated that *S. suberectus* significantly downregulated K-Ras in tumor tissues using proteomics analysis. Additionally, we found that Raf, a downstream protein, was downregulated by *S. suberectus*. Our *in vitro* and xenograft mouse study results indicate that *S. suberectus* might inhibit the K-Ras-Raf signaling pathway thereby inhibiting cell proliferation. We may further validate the relationship between K-RAS and the inhibition of cell viability induced by *S. suberectus* in the future.

In BC cells, lipogenesis is a vital process because lipids are the main constituents of the membrane. Dysregulation of FA metabolism may be a key mechanism in BC malignancy. Specifically, *de novo* lipogenesis produces the substrate essential for proliferating BC cells to regulate their membrane constitution and energetic roles in cell proliferation.^62^ Cancer cells display a “lipogenic phenotype,” which is described by increased FA synthesis and shown by the overexpression and augmented actions of enzymes related to lipogenesis.^63^^,^^64^ In the context of BC, *de novo* lipogenesis is a source of biomass and energy requirements that guard cancer cells against oxidative lipid damage.^65^ Lipid transport and absorption are critical for lipid metabolism in cancer.^66^ This feature is connected to the activation of specific tumor cellular processes, such as EMT, which is thought to aid in the development of BC and chemoresistance. Thus, inhibition of lipid synthesis is an efficient strategy for the anti-BC mechanism.^67^^,^^68^

In the present metabolomic study, we identified various metabolites associated with lipid and FA metabolism. These parameters were significantly altered during treatment with low and high doses of *S. suberectus*. As shown by PLS-DA score plots and thermograph analysis, the upregulated LysoPC (16:0), LysoPC (18:0), GDCA, and acetylcarnitine represented the initiation of FA oxidation,^69^^,^^70^ while the downregulated N-phenyl acetyl glycine indicated the inhibition of FA oxidation.^71^ Treatment with *S. suberectus* upregulated LysoPC (16:0), LysoPC (18:0), GDCA, and acetylcarnitine and downregulated N-phenyl acetyl glycine strongly indicating that *S. suberectus* promotes FA oxidation. Based on KEGG and SMPDB pathway analyses, *S. suberectus* interferes with energy metabolism in tumor tissues of mice. Based on the KEGG pathway analysis, *S. suberectus* altered various metabolic pathways such as glutamine, glyoxylate, ketone bodies, alanine, aspartate, and glutamate metabolism. Treatment with *S. suberectus* promoted β-oxidation metabolites in the tumor tissues, which was validated by the SMPDB pathway. Our outcomes are also consistent with an previous study by Huang and his team who constructed a novel pathway-driven model to investigate metabolomic information for BC diagnosis.^72^

In our study, *S. suberectus* significantly inhibited lipogenic markers such as SREBP1, LPL, FAS, HSL, ACC, and DGAT; however, treatment with *S. suberectus* promoted β-oxidation markers such as CPT1 and HMGCR. Overexpression of FA biosynthetic genes including FAS, ACC, SREBP1, and LPL have been demonstrated in several cancer phenotypes.^66^ The expression levels of ACC, FAS, and LPL were under the control of SREBP1. SREBF1 elevates ACC mRNA levels through Akt signaling and promotes various tumors.^73^ Our study showed that *S. suberectus* prevented the activation of SREBP1; thus, *S. suberectus* can be used as an anticancer drug by impeding the stimulation of SREBP1 in the lipogenic pathways of cancer cells.

FA oxidation is often controlled by numerous factors, of which AMPK is the most imperative.^74^ As a chief cellular energy sensor, activation of AMPK in cancer cells promotes energy synthesis through lipid metabolism. Antitumor research in various rodent models has confirmed that AMPK is involved in FA oxidation by directly inhibiting ACC, thereby inhibiting tumors.^75^, ^76^, ^77^ Our study also demonstrated that *S. suberectus* stimulated the activation of the AMPK signaling. In both xenograft mouse models and TNBC cells, *S. suberectus* dramatically increased the stimulation of *p*-AMPK and inhibited ACC. *S. suberectus* upregulated the *p*-AMPK activation, inhibited ACC activation, and enhanced CPT1 activation. Hence, *S. suberectus* inhibited the proliferation of BC cells by downregulating ACC and promoting fatty acid oxidation in the xenograft mice. In addition, the results of the markers ALT, AST, CREA, BUN, and pathological observations proved the safety of *S. suberectus* in the liver and kidney.^78^ The acute toxicity evaluation of our earlier *in vivo* studies indicated *S. suberectus* had an oral LD50 of 10 g/kg b. w.^9^

*S. suberectus* contains flavonoids, particularly flavanols. Catechin and its analogs are abundant in *S. suberectus*, with epicatechin, gallocatechin, and catechin appearing in descending order.^9^ Through a process of extractions, a subfraction of vine stem extracts was obtained that contained several compounds, including formononetin, daidzein, genistein, calycosin, glycyrrhizin, prunetin, epicatechin, *p*-hydroxybenzoic acid, and protocatechuic acid.^26^^,^^79^ Our laboratory has identified five isoliquiritigenin analogs from *S. suberectus* that are characterized by the methylene-bridged bischalcone, 3′,4′,5′,4″-tetramethoxychalcone, and have demonstrated significant cytotoxicity against human BC cells.^24^ Our research is currently focused on identifying the primary factors responsible for *S. suberectus*'s anti-breast cancer properties.

## 5 Conclusions

Based on this *in vitro* and *in vivo* evidence, we propose that this study represents a milestone and complements Chinese medicine. This is because *S. suberectus* inhibits lipogenesis, a hallmark of tumorigenesis in TNBC through AMPK-ACC and K-Ras-ERK signaling pathways. Further studies are underway in our laboratory to elucidate the active principles of *S. suberectus* responsible for its antitumorigenic effects.

## CRediT author statement

Xiaohui Zeng: Investigation, Validation, Formal analysis. Guowei Gong: Investigation, Methodology, Formal analysis, Validation, Data curation. Kumar Ganesan: Investigation, Methodology, Formal analysis, Validation, Data curation, Writing – original draft. Yi Wen -Investigation, Formal analysis, Validation, Data curation. Qingqing Liu: Investigation, Data curation. Juncheng Zhuo: Investigation, Methodology, Data curation. Jianming Wu: Writing – review & editing, Validation. Jiaping Chen: Conceptualization, Supervision, Project administration, Funding acquisition.

## Funding

This research was funded by the 10.13039/501100001809National Natural Science Foundation of China, China (81573663) and Guangxi Science and Technology Key 10.13039/100006190Research and Development Program, China (AB16450012).

## Ethics approval and consent to participate

All experiments were approved by the Guidelines for Laboratory Animal Care and *Committee on the Use of Live Animals in Teaching and Research* (CULATR No: 4484–17).

## Consent for publication

The manuscript is approved by all authors for publication.

## Declaration of competing interest

The authors declare that they have no known competing financial interests or personal relationships that could have appeared to influence the work reported in this paper.

## Supplementary Materials

The following is the Supplementary data to this article: Table S1: Primers used for real-time PCR. Table S2: Influence of *S. suberectus* on serum lipid metabolites in mice. Table S3. *S. suberectus* regulates liver markers in the serum of the mice.
