# Supplementary figures and images for: Spatholobus suberectus inhibits lipogenesis and tumorigenesis in triple-negative breast cancer via activation of AMPK-ACC and K-Ras-ERK signaling pathway
Source: J Tradit Complement Med. 2023 Sep 13;13(6):623–38. doi: 10.1016/j.jtcme.2023.09.002 (PMC10658394; doi:10.1016/j.jtcme.2023.09.002)

**Figure 3G**

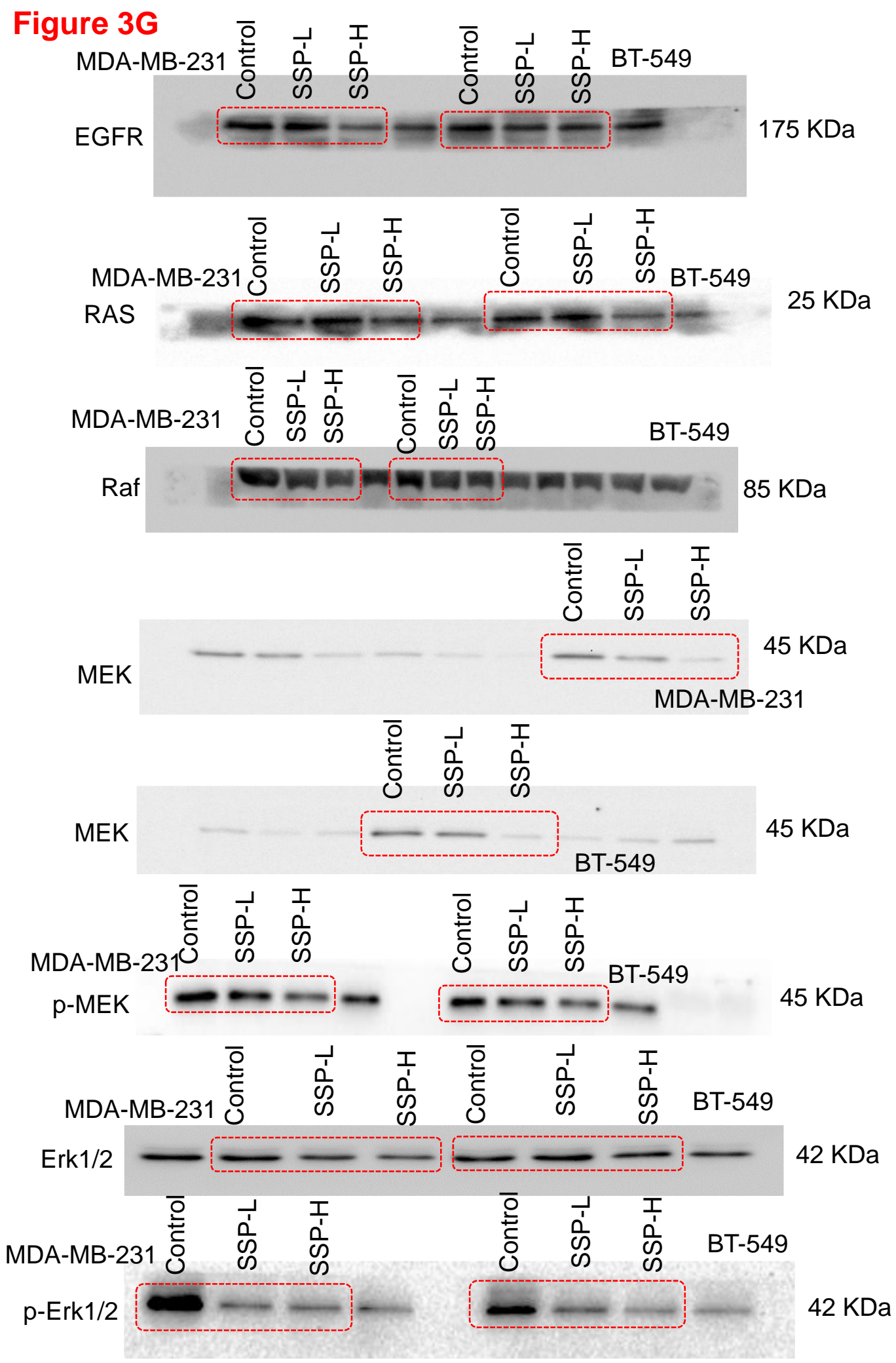

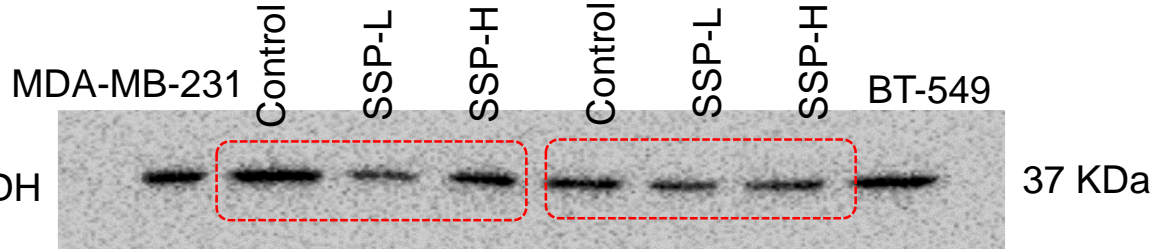

**Figure 3H**

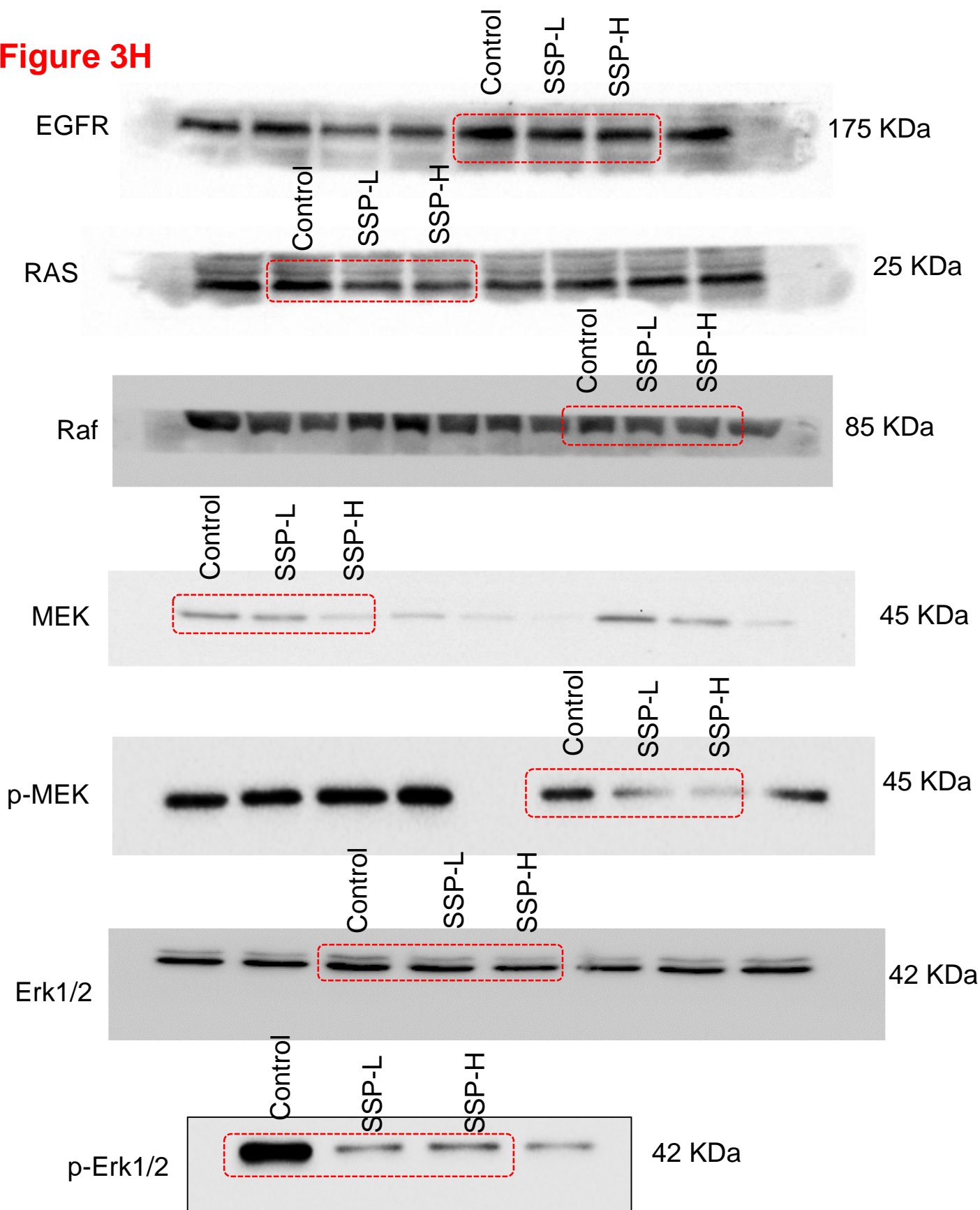

Figure 3H

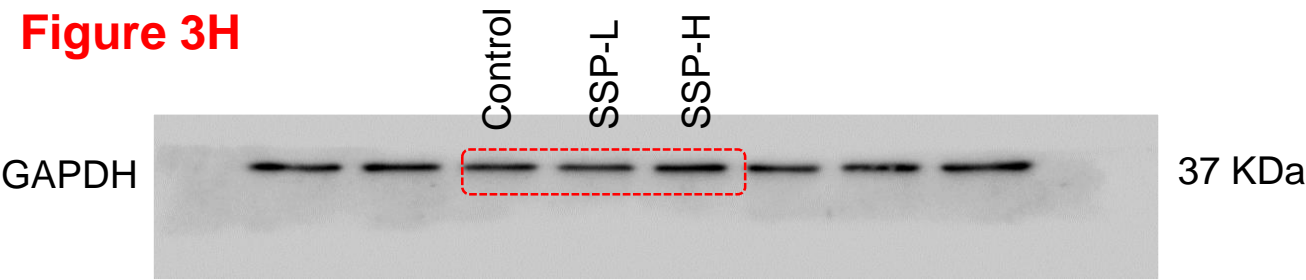

Figure 8A

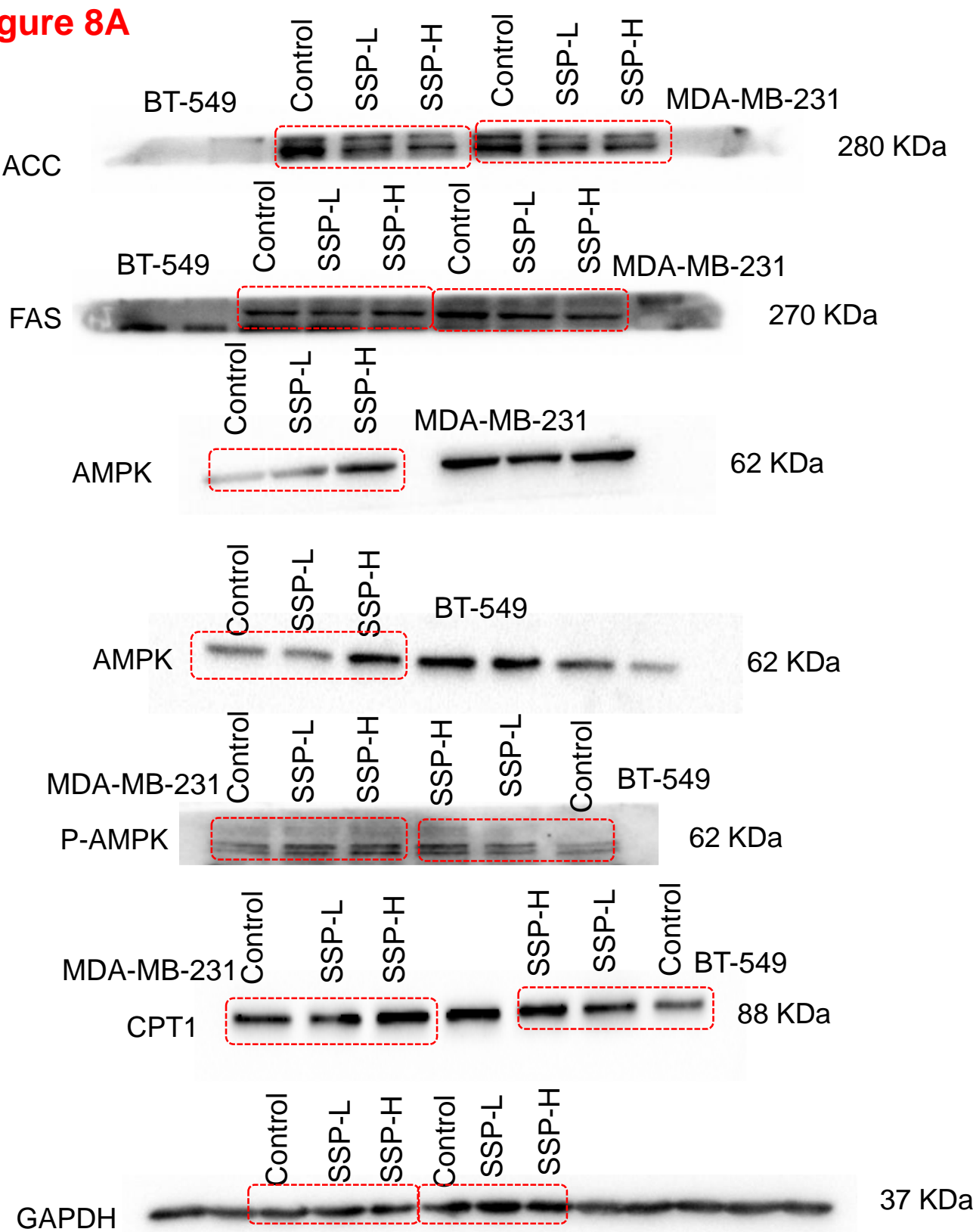

Figure 8B

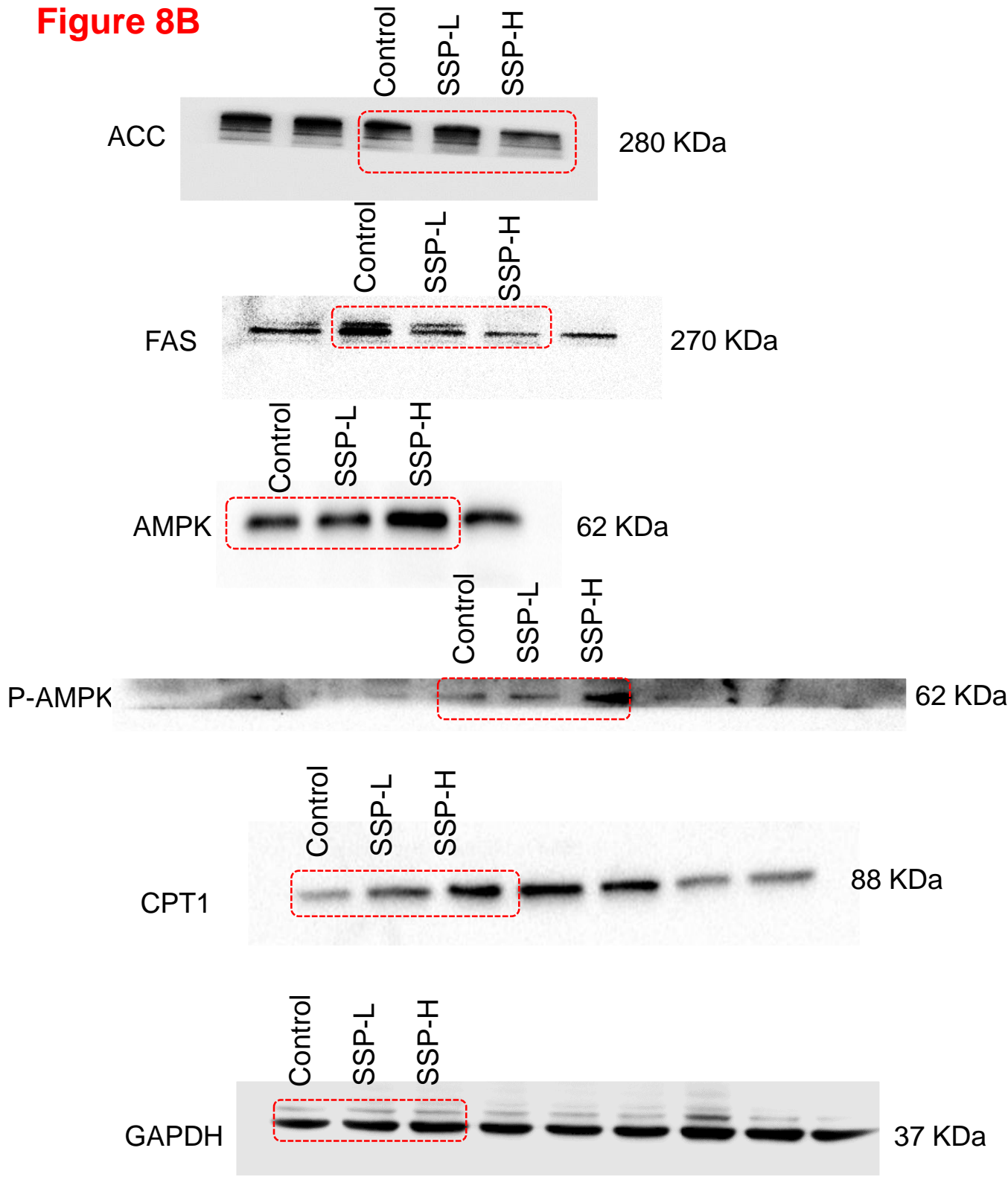

Supplement: Multimedia component 1 [file mmc1.pdf]
