# Supplementary material for: Spatholobus suberectus inhibits lipogenesis and tumorigenesis in triple-negative breast cancer via activation of AMPK-ACC and K-Ras-ERK signaling pathway
Source: J Tradit Complement Med. 2023 Sep 13;13(6):623–38. doi: 10.1016/j.jtcme.2023.09.002 (PMC10658394; doi:10.1016/j.jtcme.2023.09.002)
Supplement: Multimedia component 2 [file mmc2.docx]

**Supplementary Materials**

*Spatholobus suberectus* Inhibits Lipogenesis and Tumorigenesis in Triple-Negative Breast Cancer via Activation of AMPK-ACC and K-Ras-ERK Signaling Pathway

**Table S1: Primers used for real-time PCR**

| Target gene | Physiologic function | Former primer (5’ to 3’) | Reverse primer (5’ to 3’) |
| --- | --- | --- | --- |
| HK | Glycolysis | ACCTGTTGCCTCATTACTC | TGTTCACTTCTGGACCCT |
| PDHC | Glycolysis | CTCACGCCTATGCTAC | TCTCCCGTATGTCCTTA |
| LDHα | Glycolysis | TTGCCCTTGTTGATGT | CTGGGTTTGAGACGAT |
| G6Pase | Gluconeogenesis | CTCATCACCTTCTTCCT | GCTCACACCATCTCTT |
| SREBP1 | FA synthesis | CGCTACCGTTCCTCTATCA | CTCCTCCACTGCCACAAG |
| FAS | FA synthesis | ACTGAACTTCCGAGATA | TACGACCACGCACTA |
| ACC | FA synthesis | TGGTTGTAACGAGGTGGG | AGAGCCGATTTGGAAGGT |
| CPT1 | FA oxidation | TCAACAGCAACTACTAC | TTCCTCACGGTCTAAT |
| HSL | TG hydrolysis | GCCGATTCGCCATAGA | CTTCCCGCAGGTCATA |
| LPL | TG hydrolysis | TGGCTCCAGAGTTTGA | TTGAAGTGGCAGTTAGAC |
| IDHα | Tricarboxylic acid cycle | GACCTTCCATCTCAGCGACTTGC | TTCTGCTTGCGGATCTGCTTGTAC |
| MDH | Tricarboxylic acid cycle | ATGCTCACATAGTCCTTGGCTTGC | CACTTCCGCACTTCACTGTCTCC |
| CS | Tricarboxylic acid cycle | GTACTGAGGAAGACTGACCCT | TCCCTGGCGTAGATGACTTG |
| HMGCR | Ketone synthesis | TGACGCTCTGGTGGA | GTTACTGGGTTTGGTTTAT |
| CYP7A1 | Bile acid synthesis | TGCCGTGTTGGTGAG | TTCGCAGAAGTAGTGTAAT |
| DGAT | Diacylglycerol synthesis | GAAGAGGAGGTGCGAGAC | CCAGGATGCCATACTTGATA |
| 18S | Reference gene | ACGGCTACCACATCC | CAGACTTGCCCTCCA |

Abbreviations: ACC, Acetyl-CoA carboxylase; CPT1, Carnitine palmitoyltransferase; CS, Citrate synthase; CYP7A1, Cholesterol 7 alpha-hydroxylase; DGAT, Diacylglycerol acyltransferase; FAS, fatty acid synthase; G6Pase, glucose 6- phosphatase; HK, Hexokinase; HMGCR, 3-Hydroxy-3-Methylglutaryl-CoA Reductase; HSL, hormone sensitive lipase; IDHα, isocitrate dehydrogenase; LDHα, lactate dehydrogenease; LPL- lipoprotein lipase; MDH, malate dehydrogenase; PDHC, pyruvate dehydrogenase complex; SREBP1, sterol regulatory element-binding protein 1.

**Table S2.** **Influence of SSP on serum lipid metabolites in mice**

| **Metabolites** | **Formula** | **ID** | **Pathways** |
| --- | --- | --- | --- |
| LysoPC(16:0) | C_24_H_50_NO_7_P | HMDB0010382 | FA metabolism |
| LysoPC(18:0) | C_26_H_54_NO_7_P | HMDB0010384 | FA metabolism |
| GCDA | C_26_H_43_NO_5_ | HMDB0000637 | Bile acid metabolism |
| N-phenyl acetyl glycine | C_10_H_11_NO_3_ | HMDB0000821 | Beta-oxidation |
| L-Acetylcarnitine | C_9_H_17_NO_4_ | HMDB0000201 | Beta-oxidation |
| L-Glutamic acid | C_5_H_9_NO_4_ | HMDB0000148 | D-Glutamine metabolism |
| 3-hydroxybutyric | C_4_H_8_O_3_ | HMDB0000357 | Butanoate metabolism |
| AA | C_6_H_6_O_6_ | HMDB0000072 | Citrate cycle |
| FMA | C_4_H_4_O_4_ | HMDB0000134 | Citrate cycle |
| CA | C6H8O7 | HMDB0000094 | Citrate cycle |

The potential metabolites with VIP > 1 and p < 0.05 were selected as potential biomarkers.

Abbreviations: AA, acotinic acid; CA, citric acid; FMA, fumaric acid; GCDA, glycochenodeoxycholate

**Table S3.** **SSP regulates liver markers in the serum of the mice**

|  | CREA(umol/L) | BUN(mmol/L) | ALT(IU/L) | AST(IU/L) |
| --- | --- | --- | --- | --- |
| Control | 51.48±3.40 | 4.83±0.66 | 18.28±7.57 | 33.75±16.61 |
| SSP-H | 49.95±6.78 | 5.45±0.45 | 22.88±6.30 | 45.78±19.67 |
| SSP-L | 51.63±8.24 | 5.26±0.81 | 19.26±6.62 | 33.41±7.32 |

Abbreviations: CREA, creatinine; BUN, blood urea nitrogen; ALT, alanine transaminase; AST, aspartate transaminase
